# ^1^H NMR Spin-Lattice Relaxometry of Cement Pastes with Polycarboxylate Superplasticizers

**DOI:** 10.3390/ma13245626

**Published:** 2020-12-10

**Authors:** Min Pang, Zhenping Sun, Qi Li, Yanliang Ji

**Affiliations:** 1 Key Laboratory of Advanced Civil Engineering Materials, Ministry of Education, Tongji University, Shanghai 201804, China; pangmin@tongji.edu.cn (M.P.); yanliangji@tongji.edu.cn (Y.J.); 2 School of Materials Science and Engineering, Tongji University, Shanghai 201804, China; liqi_article@126.com

**Keywords:** nuclear magnetic resonance, spin-lattice relaxometry, proton, hydration kinetics, superplasticizer

## Abstract

^1^H spin-lattice relaxometry (T_1_, longitudinal) of cement pastes with 0 to 0.18 wt % polycarboxylate superplasticizers (PCEs) at intervals of 0.06 wt % from 10 min to 1210 min was investigated. Results showed that the main peak in T_1_ relaxometry of cement pastes was shorter and lower along with the hydration times. PCEs delayed and lowered this main peak in T_1_ relaxometry of cement pastes at 10 min, 605 min and 1210 min, which was highly correlated to its dosages. In contrast, PCEs increased the total signal intensity of T_1_ of cement pastes at these three times, which still correlated to its dosages. Both changes of the main peak in T_1_ relaxometry and the total signal intensity of T_1_ revealed interferences on evaporable water during cement hydration by dispersion mechanisms of PCEs. The time-dependent evolution of weighted average T_1_ of cement pastes with different PCEs between 10 min and 1210 min was found regular to the four-stage hydration mechanism of tricalcium silicate.

## 1. Introduction

Since Bloch [1] and Purcell [2] awarded the Nobel Prize for successful monitoring the magnetic situation of protons in water and parafilm using low-field nuclear magnetic resonance(NMR) instruments, ^1^H NMR (proton NMR) has been used as an effective technology in cement and concrete research for a long while. ^1^H NMR could detect the nuclear spin-lattice relaxometry (T_1_, longitudinal) or spin–spin relaxometry (T_2_, transverse) of ^1^H nuclei. T_1_ and T_2_ depend on fluctuations in magnetic dipole–dipole interactions caused by the relative motion of pairs of spins [3]. Relaxometry (relaxation time) may extend due to the relative motion of spins in a fluid as water or oil, theoretical studies have been done by Korb [4,5] previously, and recent studies on cement hydrates by McDonald [6,7,8,9].

There are many studies about water transport and distributions in cement-based materials based on T_2_ relaxometry [10,11,12,13]. T_2_ relaxometry has also been used for detecting porosity of oil-well cement pastes [14], C_3_S hydrated pastes [15,16], white cement mortars [17], lime plaster–brick systems [18], complex wall materials [19], carbonated cement pastes [20]. Besides macropores in cement-based materials, nanopores (or gel pores) in calcium silicate hydrate (C-S-H) gel could also be demonstrated by T_2_ relaxometry [21]. Given that C-S-H gel being one poorly crystalline, which is quasi-amorphous and contains nanopores with water [22], some attempts have been made with water in C-S-H gel pores [23,24]. There are some other applications of T_2_ relaxometry in alkali-activated binders [25], lime concrete [26], cement pastes with superabsorbent polymers [27], cellulose ethers [28], woods [29], MgO-based cement [30]. However, the paramagnetic species (mainly Fe^3+^) in cement pastes could influence T_2_ relaxometry [6]. It has been reported that T_2_ relaxometry would result in a reduced volume because of its access to the paramagnetic species in a single channel based on the crystalline structure of ettringite [31].

Compared to T_2_ relaxometry, there are few applications of T_1_ relaxometry in cement-based materials. It has been found that T_1_ relaxometry of white cement pastes with w/c = 0.3, 0.4, 0.6, 0.7 could show a significant increase after drying at 105 °C while the freeze–thaw cycling of 25 times could not make obvious changes [32]. Many theoretical studies of T_1_ relaxometry in cement-based materials have been done by one joint research group in Yugoslavia and Canada from 1978 to 1996. They have measured the T_1_ relaxometry of absorbed water in cement pastes and C_3_S pastes during the hardening process [33]. A method has been proposed to determine the specific surface of cement hydrates based on T_1_ relaxometry (1/T_1_) [34]. T_1_ relaxometry of Portland cement and white cement, as well as white cement with 5 wt % MSF salts (sulfonated-melamine-formaldehyde), has been monitored, respectively [35,36,37]. They have also compared signal differences between the synthesized white cement at 40 MHz and at 200 MHz [38]. The fractal geometry of C-S-H gel, quantitative changes of water and Ca(OH)_2_ in white cement pastes have been monitored successively [39,40]. T_1_ relaxometry of calcium aluminate cement, T_1_ relaxometry of self-stressed expansive cement, T_1_-weighted line shape of white cement pastes have been explored by some coworkers with this group afterward [41,42,43]. Moreover, some researchers have tried to correlate signals of T_1_ relaxometry to microcracks in cement pastes [44]. Other researchers have studied the dynamics of liquid water in pores of cement-based materials based on T_1_ relaxometry [45].

As it has been found that 5 wt % MSF salts could change T_1_ relaxometry of white cement pastes, which have acted as the superplasticizers [37], could polycarboxylate superplasticizers (PCEs) change T_1_ relaxometry of Portland cement pastes? In this research, effects of the synthesized PCEs on the hydration process of Portland cement pastes were initially explored by ^1^H NMR technique based on T_1_ relaxometry and its total signal intensity at three selected time points of 10 min, 605 min, 1210 min, as well as the evolution process of weighted average T_1_ from 10 min to 1210 min. We are certain that this study would enrich the applications of T_1_ relaxometry in cement-based materials. PCEs are popular chemicals for ultra-high performance concrete (UHPC). Hence, this study could also provide useful data to enrich understandings of the effects of PCEs to cement pastes in UHPC.

## 2. Materials and Methods

### 2.1. Materials

The P.Ⅱ.52.5 cement used in this experiment was purchased from Jiangnan Onoda Cement Co. LTD., Jiangsu, China. The fineness of cement was 315 m^2^/kg, and its chemical composition as supplied by its manufacturer is shown in Table 1. The PCEs used in this experiment were synthesized from these materials. Methyl allyl polyethenoxy ether (TPEG) was purchased from Yangzi Aoke Chemical Company(Nanjing, China). Maleic anhydride (MA, analytical-grade), acrylic acid methyl ester (AAME, analytical-grade) and acrylic acid (AA, analytical-grade) were purchased from Shanghai Guoyao Chemical Company (Shanghai, China).

### 2.2. Preparation of PCEs

**Figure 1:**
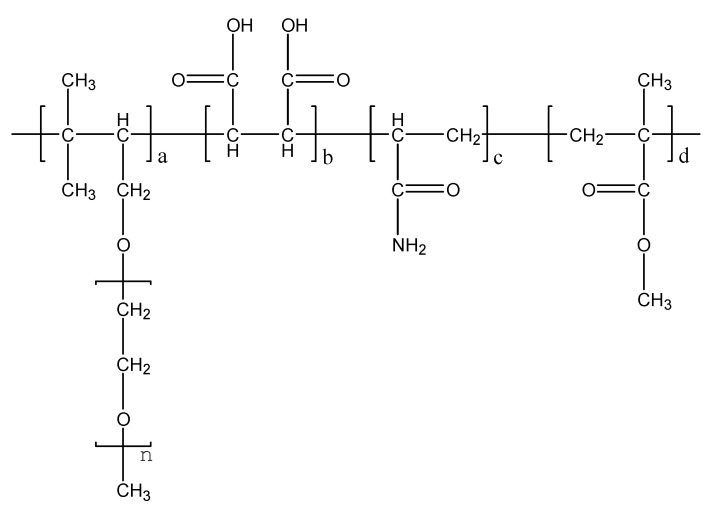
The synthesized formula of polycarboxylate superplasticizers (PCEs).

PCEs were synthesized via aqueous free radical copolymerization at the molar ratios of TPEG: MA: AAME: AA at 6:9:12:6. The average polymerization degree of TPEG was 35, and the Mn of TPEG was 2400 g/mol, which were provided by its manufacturer. The equipment for the synthesized process of PCEs was as same as the previous investigation [46]. The initiator in the synthesized process was (NH_4_)_2_S_2_O_8_ (ammonium persulfate, AP), which was 3 wt % to total monomers and purchased from Shanghai Guoyao Chemical Company (Shanghai, China). The temperature in the synthesis process was 80 °C. The adding time of monomers and the soaking time in the synthesized process were 1 h and 0.5 h, respectively. The synthesized formula is shown in Figure 1.

### 2.3. NMR Equipment and Theory

The low-field NMR instrument in this experiment was PQ-001 NMR (Niumag Electric Corporation, Shanghai, China). This instrument had a constant magnetic field of 0.49 T, a proton resonance frequency of 21 MHz, a permanent magnet of 32 °C. T_1_ relaxometry was detected with the inversion recovery (IR). The IR sequence (π-τ-π/2-acq) was applied to measure the T_1_, where t was the waiting time, acq was the received signal. After the NMR equipment debugged, the prepared samples of different cement pastes with PCEs were poured into an NMR tube with a height between 17 mm to 20 mm and then sealed by a PTFE film.

Commonly, the T_1_ relaxometry used in the cement-based materials was based on the fast-exchange model, which was originated from the standard model. Based on the assumption that the molecular exchanging between two phases faster than individual proton relaxation times, the integrated relaxation rate could be described as Equation (1) in [47]. In Equation (1), $T_{1,2}^{\mathrm{bulk}}$ and $T_{1,2}^{\mathrm{surf}}$ were the proton relaxation times in the bulk and at the surface, $f_{\mathrm{surf}}$ and $f_{\mathrm{bulk}}$ were the volume fractions of the surface and bulk phases. The correlation between $f_{\mathrm{surf}}$ and $f_{\mathrm{bulk}}$ could be described as Equation (2). The surface relaxation rate was much higher than the bulk relaxation rate, so Equation (1) was simplified to Equation (3), which had been successfully used [6]. In Equation (3), $\rho_{1,2}$ was the corresponding surface relaxivity, S and V were the pore surface and the pore volume.

(1) $$\frac{1}{T_{1,2}}=\frac{f_{\mathrm{bulk}}}{T_{1,2}^{\mathrm{bulk}}}+\frac{f_{\mathrm{surf}}}{T_{1,2}^{\mathrm{surf}}}$$

(2) $$f_{\mathrm{surf}}+f_{\mathrm{bulk}}=1$$

(3) $$\frac{1}{T_{1,2}}=\rho_{1,2}\frac{S}{V}+\frac{1}{T_{1,2}^{\mathrm{bulk}}}\approx \rho_{1,2}\frac{S}{V}$$

### 2.4. Methods

**Figure 2:**
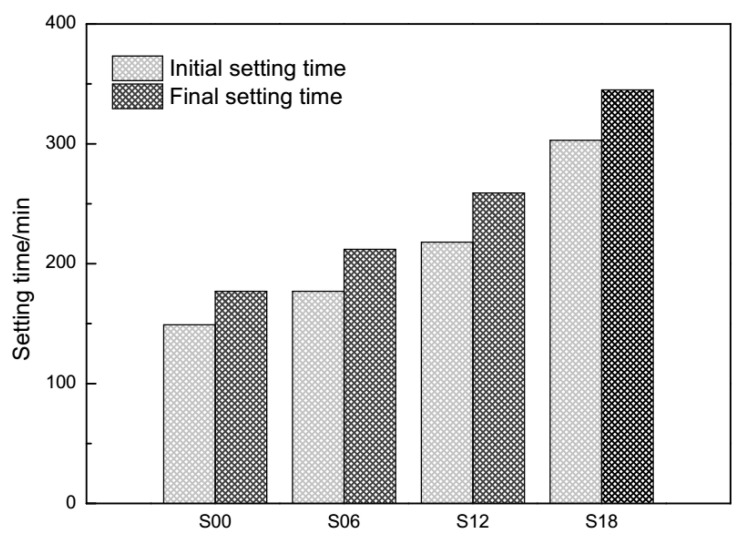
Effects of the synthesized PCEs on the setting time of cement pastes.

The water-to-cement ratio (w/c) was 0.28, with the detailed proportions listed in Table 2. In cement pastes with PCEs, the spectroscopic observation of the liquid water phase was achieved by exploiting T_1_ relaxometry from the initial time point at 10 min, with intervals of 30 min, to the final time point at 1210 min. The curves of T_1_ relaxometry were obtained by fitting the magnetization recovery curves with a log-normal distribution of relaxation times. The setting time of cement pastes with PCEs is shown in Figure 2, which was according to GB/T 1346–2011. It could be seen that the setting time of cement pastes were delayed by PCEs.

## 3. Results and Discussion

**Figure 3:**
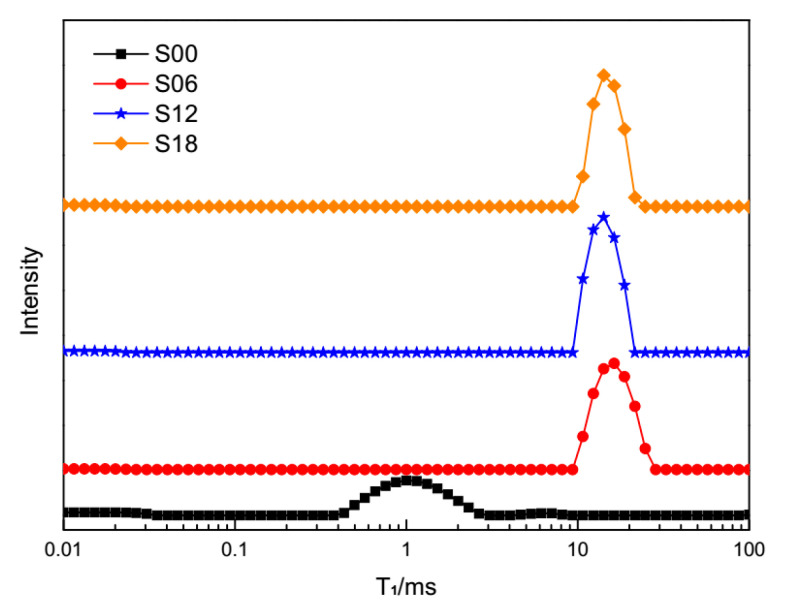
T_1_ relaxometry of different cement pastes at 10 min.

**Figure 4:**
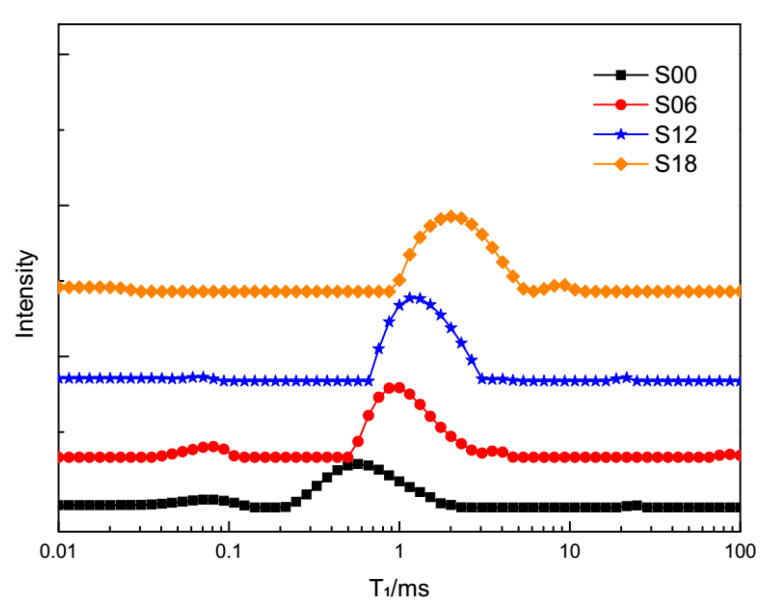
T_1_ relaxometry of different cement pastes at 605 min.

**Figure 5:**
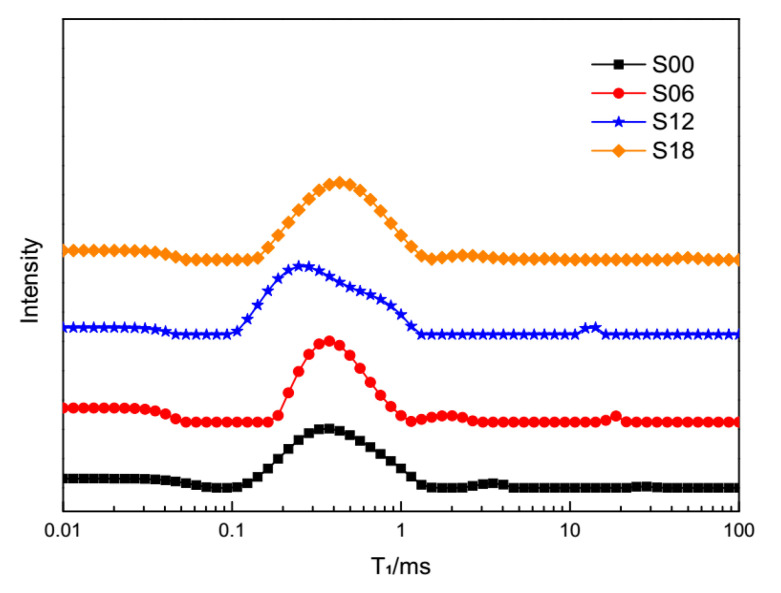
T_1_ relaxometry of different cement pastes at 1210 min.

T_1_ relaxometry of cement pastes with PCEs at 10 min, 605 min and 1210 min are shown in Figure 3, Figure 4 and Figure 5, respectively. One can see that there is a main peak in every curve of T_1_ relaxometry. To be specific, for T_1_ relaxometry at 10 min in Figure 3, the main peak of plain cement pastes (S00) is shorter than those of cement pastes with PCEs. Among T_1_ relaxometry of cement pastes with PCEs (S06, S12, S18), the main peak seems to stand at the same time. Furthermore, the main peak of plain cement pastes is obviously lower than those of cement pastes with PCEs. The main peak of S06 (0.06 wt % PCEs) is remarkably lower than those of S12 (0.12 wt % PCEs) and S18(0.18 wt % PCEs), while those of S12 and S18 are nearly the same.

Based on principles of the fast-exchange model [47], any shift of peaks in T_1_ relaxometry means changes in the motion trail of a proton (or water). T_1_ relaxometry of plain cement pastes (S00) represents the “normal” motion trails of protons in cement pastes at 10 min. Consequently, the prolonged main peaks of cement pastes with PCEs (S06, S12, S18) reveal that the “normal” motion trails of the protons have already been hindered by the dispersed cement grains due to PCEs.

According to [40,43], this main peak represents the quantity of evaporable water in cement pastes. The lowest main peak of plain cement pastes (S00) equals the smallest quantity of evaporable water left in this sample. The higher main peaks of the cement pastes with PCEs (S06, S12, S18) mean that more evaporable water was left in those samples. In other words, the hydration process in plain cement pastes (S00) exhausted more evaporable water than cement pastes with PCEs.

It can be seen that the main peaks of cement pastes with PCEs (S06, S12, S18) in Figure 4 are all lower and shorter than those in Figure 3. The deepening hydration process is becoming an extensive consumer of evaporable water. Meanwhile, the conglomerating cement hydrates are becoming strong inhibitors of the “normal” motion trail of protons. All of the main peaks of cement pastes (S00, S06, S12, S18) in Figure 5 are shorter than those in Figure 4. As shown by the setting time of cement pastes with PCEs in Figure 2, the hardened cement pastes at 1210 min have controlled the “normal” motion trail of the protons. Larger area ratios of the main peak in Figure 5 may refer to the bleeding situation in some subregions in hardening cement pastes, which has been revealed by T_1_ relaxometry [48].

**Figure 6:**
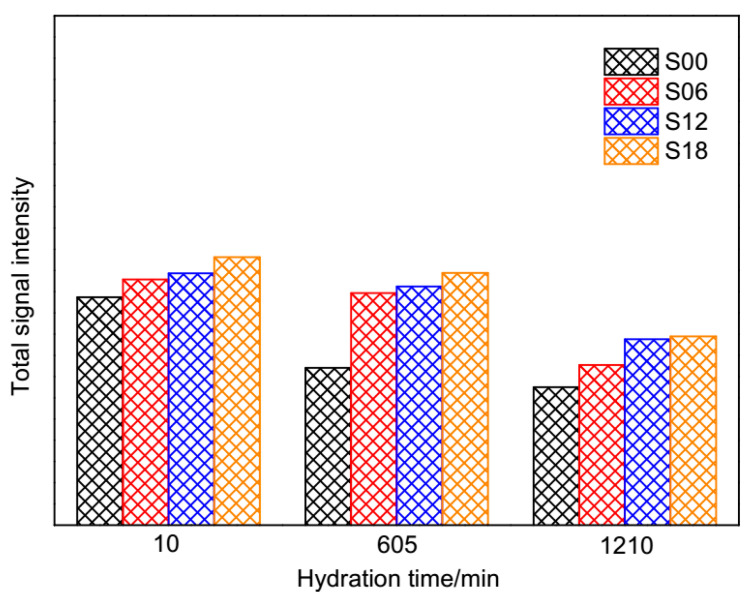
The total signal intensity of cement pastes at different hydration times.

The total signal intensity of T_1_ is proportional to the quantity of evaporable water in cement pastes [49]. It can be found in Figure 6 that the total signal intensity was decreasingly lower from 10 min to 1210 min, which implies the downside of evaporable water in cement pastes. The orders of evaporable water left in cement pastes at three times are opposite to the dosages of PCEs.

**Figure 7:**
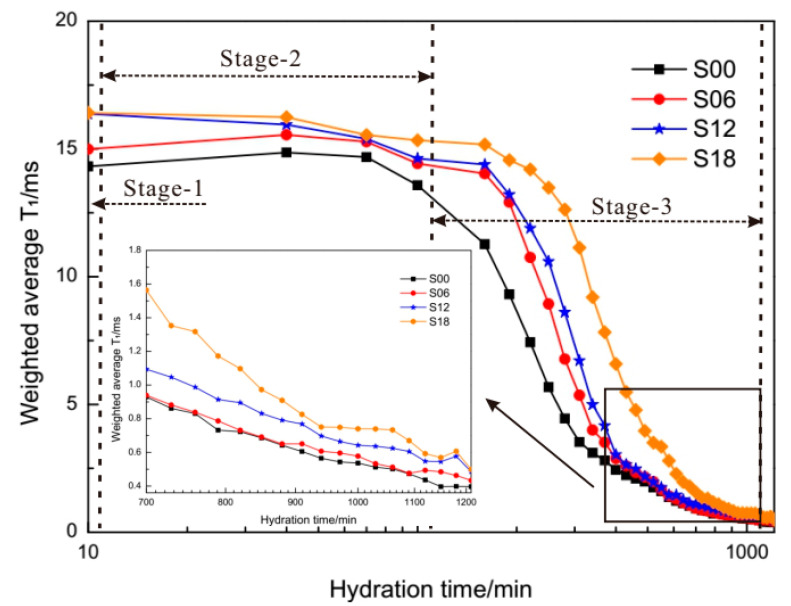
Time-dependent evolution of weighted average T_1_ of different cement pastes within 1210 min.

The time-dependent evolution of weighted average T_1_ is shown in Figure 7. It is shown that the time evolution of the weighted average T_1_ of cement pastes with PCEs has decreased successively. The time-dependent evolution of T_1_ has been described according to the four-stage hydration mechanism of tricalcium silicate (3CaO.SiO_2_, C_3_S), which occupies 50 wt % to 70 wt % in clinkers [50].

The first stage of C_3_S hydration is the initial period, which is within 15 min. The initial period in Figure 7 is only 5 min. During the initial period, the original hydration products form gelatinous coatings surrounding cement grains. The entire proton magnetization relaxes with a common T_1,_ which is due to the fast exchange between water spins in the various environments. The fast exchange may maintain the apparent relaxation homogeneity because of the fluidity of the gelatinous coating and the permeability of the gel–liquid interface [35]. Therefore, the weighted average T_1_ of each sample (S00, S06, S12, S18) seems unchanged from 10 min to 15 min in Figure 7. There are still some differences among the initial values of weighted average T_1_ at 10 min. The order of these initial values is against the dosages of PCEs. The order of weighted average T_1_ at 10 min is the feedback to gelatinous coatings thickness of cement hydrates, which has means that the coatings of hydrates in plain cement pastes were the thickest.

The second stage of C_3_S hydration is the slow-reaction (dormant) period, which is from 15 min to 120 min. During the dormant period, the weighted average T_1_ of each sample (S00, S06, S12, S18) decreased slowly (Figure 7). The fast exchange of water was hindered by the newly formed hydrates coatings. Therefore, the decline of weighted average T_1_ occurred, which was shown by comparisons of proton magnetization fractions and relaxation times of H_2_O, Ca(OH)_2_ and C-S-H gel [40]. The third stage of C_3_S hydration is the accelerated period, which is from 120 min to 1210 min. During the accelerated period, the weighted average T_1_ of each sample (S00, S06, S12, S18) has decreased sharply. The fast exchange of water was heavily hindered by thickening hydrates coatings. The weighted average T_1_ of each sample may be regarded as the reverse translation of resistance to the fast exchange of water.

Why does the weighted average T_1_ of S18 (most PCEs) have the biggest value during the whole timeline? Based on T_2_ relaxometry of cement pastes with PCEs [51], dispersion mechanism of PCEs in OPC [52], the effects of PCEs on C_3_A/gypsum [53], the bleeding condition of fresh cement pastes caused by PCEs [54], this answer would come from two aspects: (1) retardation of PCEs on cement hydration; (2) dispersion effects of PCEs on cement grains.

## 4. Conclusions

(1) The main peak in the T_1_ relaxometry of cement pastes at the hydration times of 10 min, 605 min and 1210 min was delayed by polycarboxylate superplasticizers (PCEs). The delayed intensity correlated to the dosage of PCEs. The main peak in T_1_ relaxometry of cement pastes became shorter along with the hydration times from 10 min to 1210 min;
(2) The height of the main peak in T_1_ relaxometry of cement pastes at these three times was decreased by PCEs. In addition to the larger area ratios of the main peak in T_1_ relaxometry of cement pastes at the hydration time of 1210 min due to bleeding, the decreased intensity correlated to the dosage of PCEs:
(3) The main peak in T_1_ relaxometry of cement pastes represented the quantity of evaporable water in cement pastes. The delaying situation and the decreasing situation of the main peak was due to the dispersion mechanism and the retardation mechanism of PCEs on cement grains;
(4) The total signal intensity of T_1_ of cement pastes at these three times was increased by PCEs. The increasing intensity correlated to the dosage of PCEs. The total signal intensity of T_1_ of cement pastes became smaller during the hydration process. As this intensity was proportional to the quantity of evaporable water, its changes mirrored disturbances of PCEs to situations of evaporable water in the hydration process;
(5) The time-dependent evolution of weighted average T_1_ of cement pastes from 10 min to 1210 min was elevated by PCEs. The elevated intensity correlated to the dosage of PCEs. The curves of weighted average T_1_ of cement pastes were well followed by the four-stage hydration mechanism of tricalcium silicate.

## Figures and Tables

**Table 1:** Chemical compositions of cement (wt %).

	SiO_2_	CaO	Al_2_O_3_	Fe_2_O_3_	MgO	Na_2_O	K_2_O	TiO_2_	SO_3_	Loss on Ignition
Cement	21.1	64.3	5.3	2.6	1.7	0.2	0.35	0.3	1.7	2.45

**Table 2:** Mix proportion of cement pastes with PCE (wt %).

Sample	Cement (g)	Water (g)	PCE (g)
S00	1	0.28	0
S06	1	0.28	0.06
S12	1	0.28	0.12
S18	1	0.28	0.18
